# Supplementary material for: Synthesis and Self-Assembly of Hyperbranched Multiarm Copolymer Lysozyme Conjugates Based on Light-Induced Metal-Free Atrp
Source: Nanomaterials (Basel). 2023 Mar 11;13(6):1017. doi: 10.3390/nano13061017 (PMC10053904; doi:10.3390/nano13061017)
Supplement: Supplementary file 1 [file nanomaterials-13-01017-s001.zip › nanomaterials-2268786-supplementary.pdf]

Supporting Information

Synthesis and self-assembly of hyperbranched multiarm copolymer lysozyme conjugates based on light-induced metal-free ATRP

Jianguo Yi, Yan Qin, Yue Zhang*

Characterization: ^{1}H NMR measurements were carried out on a 400 MHz Varian UNITY-plus NMR spectrometer. The M_n and the dispersity were measured by a Hitachi gel permeation chromatography (GPC) with DMF as the mobile phase and PMMA as the standards. The GPC was equipped with a Hitachi L-2490 refractive index detector and a Viscotek 270 dual detector. Scanning electron microscopy (SEM) was measured with an FEI Apreo S LoVac electron microscope. The hydrodynamic diameters (D_h), the polydispersities (PDI) of the assemblies were measured by a Malvern Zetasizer Nano-ZS. LCST and lysozyme activity were determined on a Shimadzu UV-2450 spectrophotometer. The farultraviolet circular dichroism (CD) spectra were collected on a JASCO J-815 spectrometer equipped with a thermally regulated cell compartment. The measurements were performed in a quartz cuvette with an optical path of 1 mm. The scanning range was from 190 to 250 nm and the scanning speed was 100 nm min^{-1}.

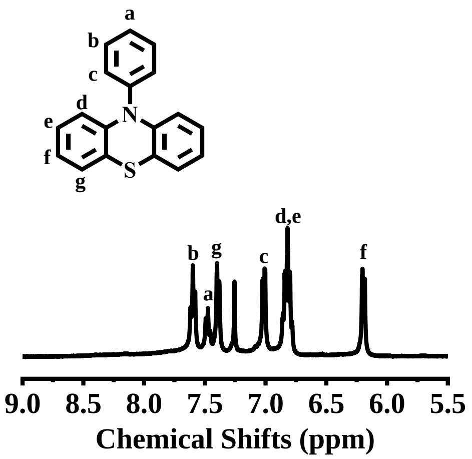

Figure S1. ^{1}H NMR spectrum of PTH in $CDCl_3$

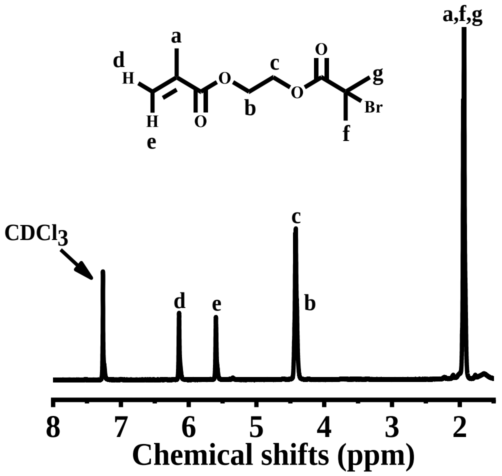

Figure S2. ^{1}H NMR spectrum of BMA in $CDCl_3$

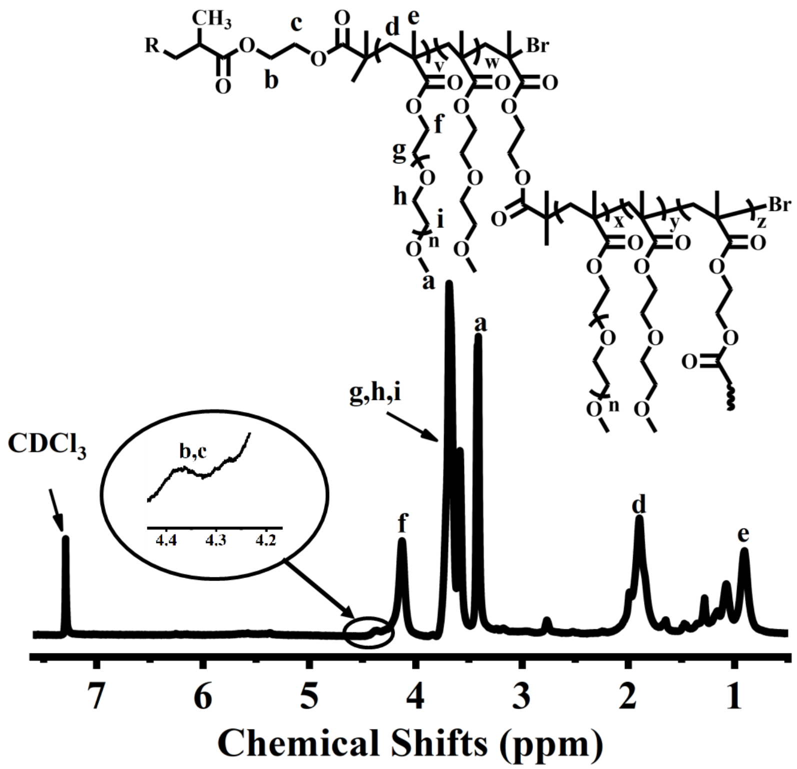

Figure S3. ^{1}H NMR spectrum of h_2P(DEGMA-co-OEGMA) in $CDCl_3$

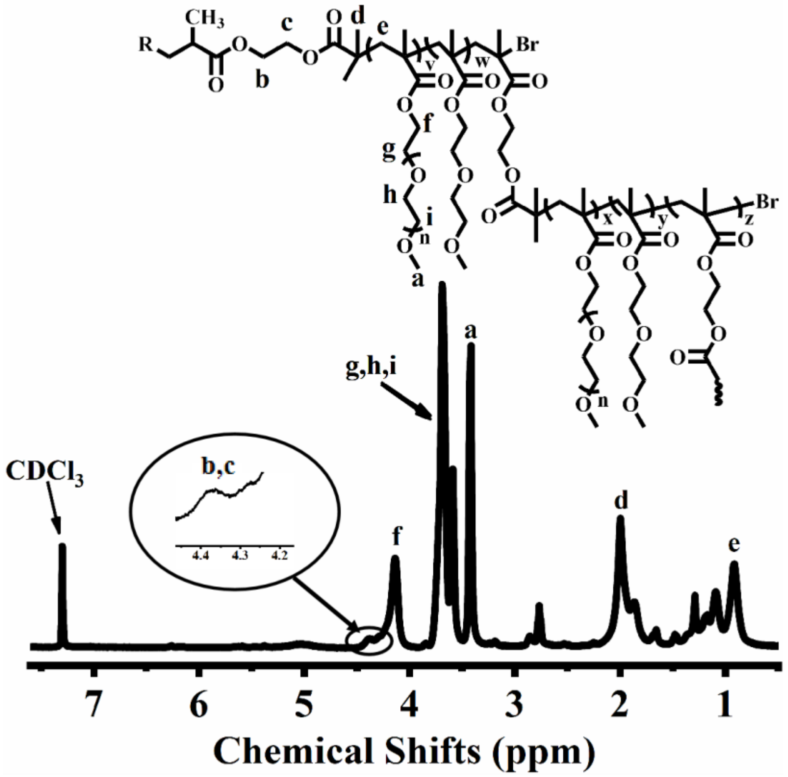

Figure S4. ^{1}H NMR spectrum of h_3P(DEGMA-co-OEGMA) in $CDCl_3$

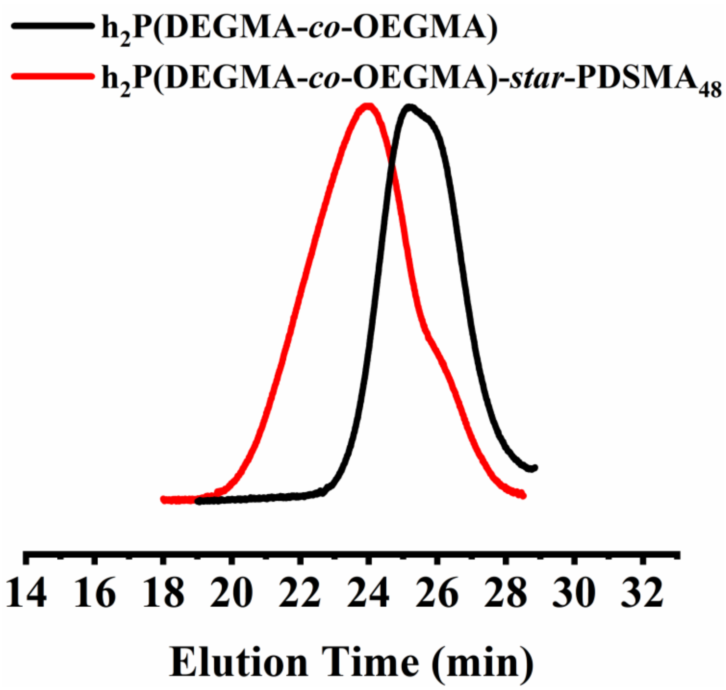

Figure S5. GPC curves of h_2P(DEGMA-*co*-OEGMA) (black line) and h_2P(DEGMA-*co*-OEGMA)-*star*-PDSMA$_{48}$ (red line)

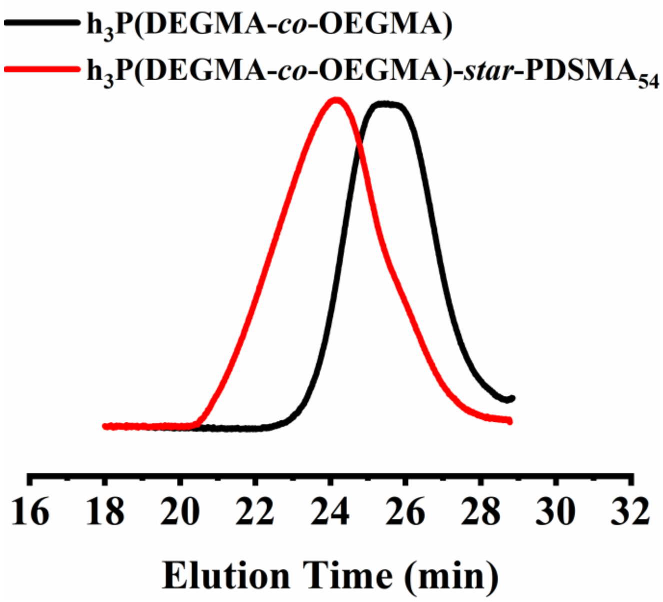

Figure S6. GPC curves of h_3P(DEGMA-*co*-OEGMA) (black line) and h_3P(DEGMA-*co*-OEGMA)-*star*-PDSMA$_{54}$ (red line)

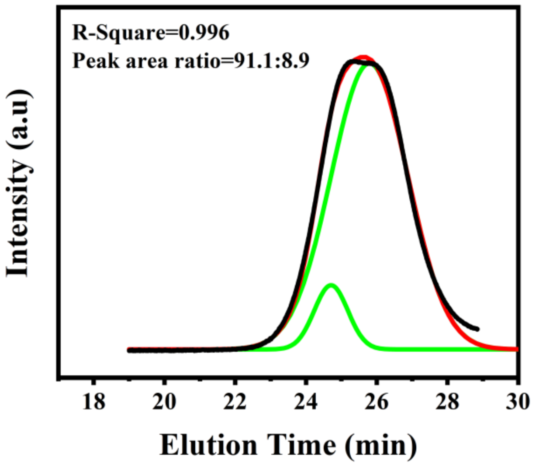

Figure S7. Fitting result of the GPC curve of h_1P(DEGMA-*co*-OEGMA). The M_ns of the two peaks are 12.2 and 24.1 kDa.

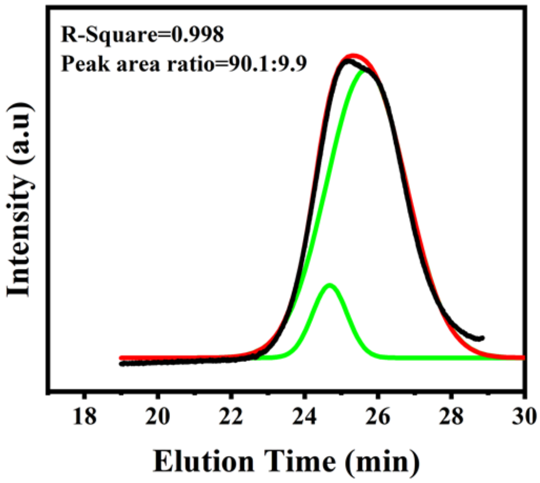

Figure S8. Fitting result of the GPC curve of h_2P(DEGMA-*co*-OEGMA). The M_ns of the two peaks are 13.3 and 24.2 kDa.

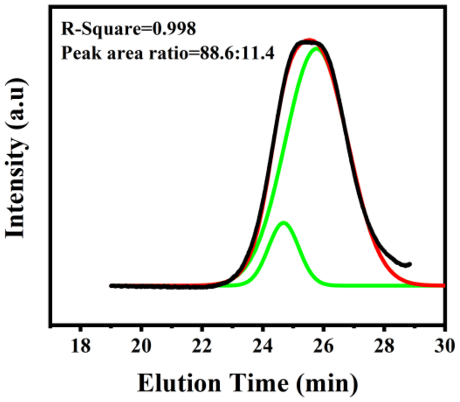

Figure S9. Fitting result of the GPC curve of h_3P(DEGMA-*co*-OEGMA). The M_ns of the two peaks are 12.5 and 24.3 kDa.

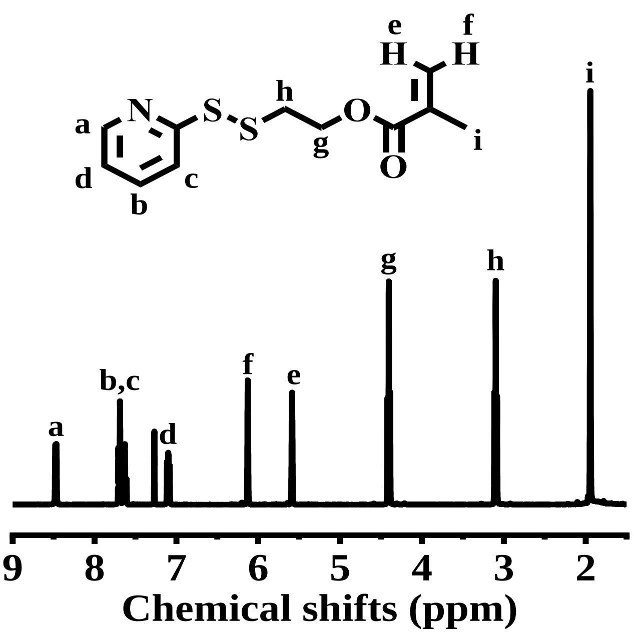

Figure S10. ^{1}H NMR spectrum of DSMA in $CDCl_3$

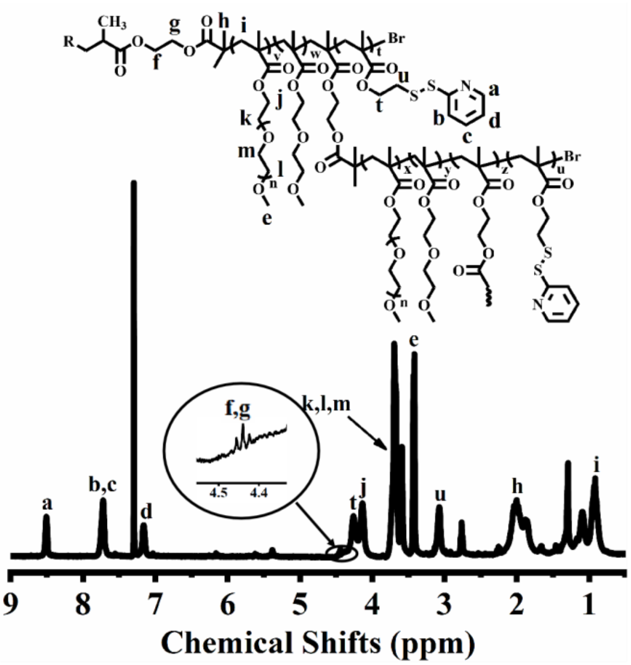

Figure S11. ^{1}H NMR spectrum of h_2P(DEGMA-*co*-OEGMA)-*star*-$PDSMA_{48}$ in $CDCl_3$

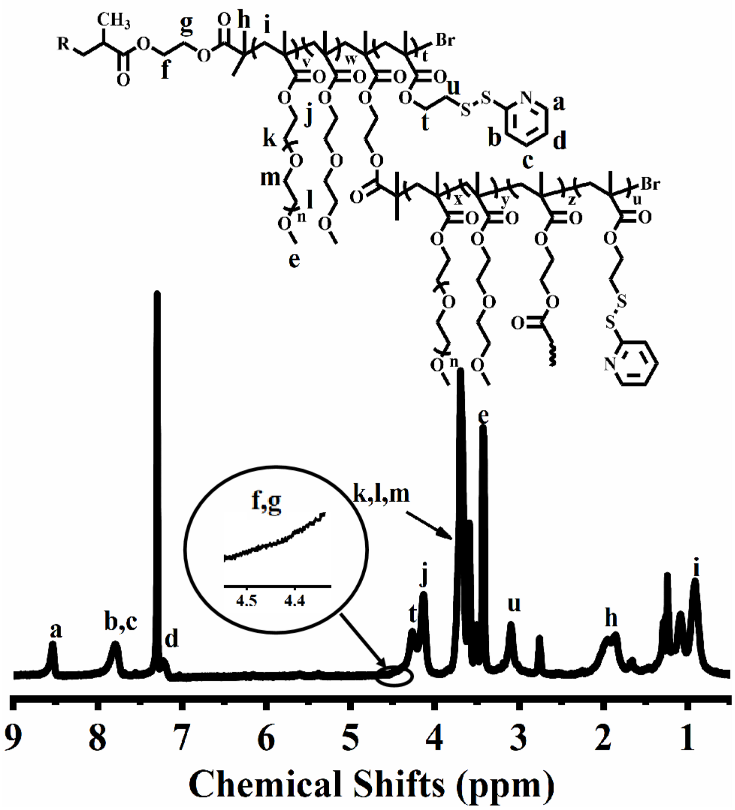

Figure S12. ^{1}H NMR spectrum of h_3P(DEGMA-*co*-OEGMA)-*star*-PDSMA$_{54}$ in $CDCl_3$

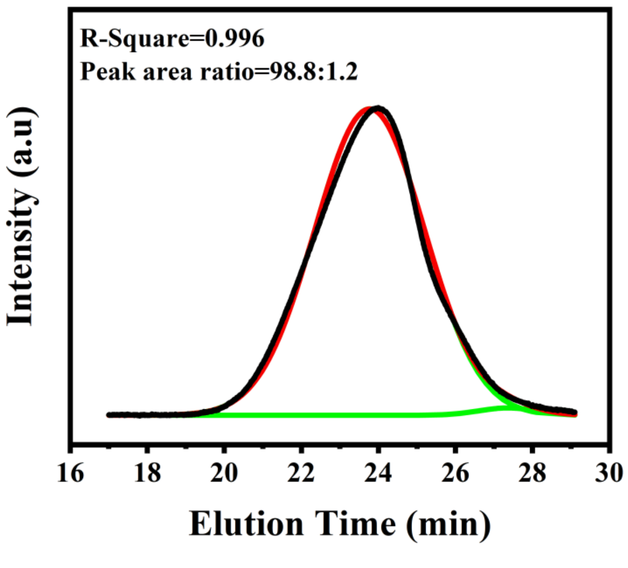

Figure S13. Fitting result of the GPC curve of h_1P(DEGMA-*co*-OEGMA)-*star*-PDSMA$_{45}$. The M_ns of the two peaks are 5.1 and 35.1 kDa.

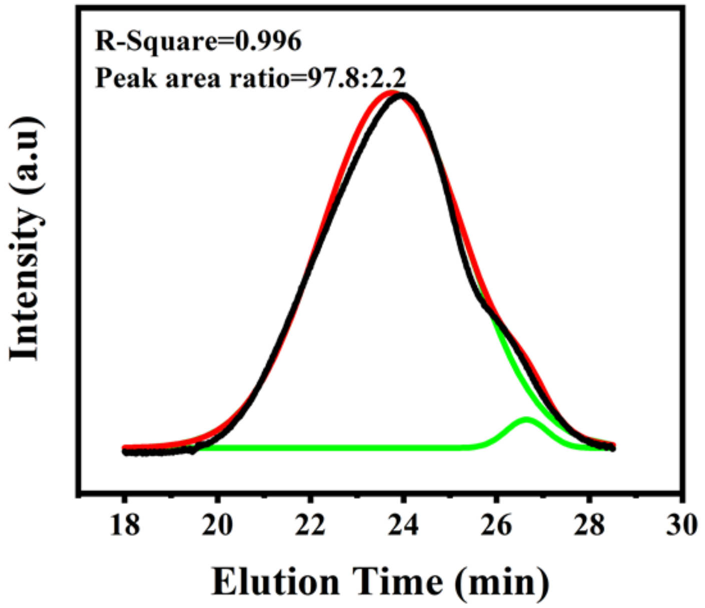

Figure S14. Fitting result of the GPC curve of h_2P(DEGMA-*co*-OEGMA)-*star*-$PDSMA_{48}$. The M_ns of the two peaks are 7.2 and 34.5 kDa.

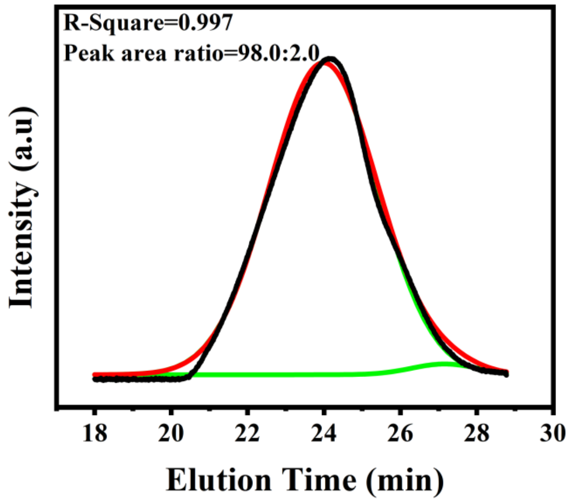

Figure S15. Fitting result of the GPC curve of h_3(DEGMA-*co*-OEGMA)-*star*-$PDSMA_{54}$. The M_ns of the two peaks are 5.2 and 35.3 kDa.

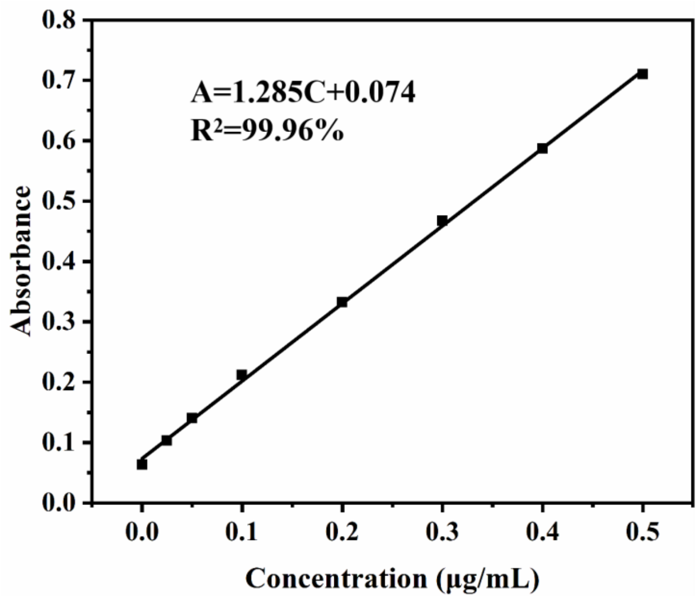

Figure S16. Standard curve of lysozyme determined by BCA method

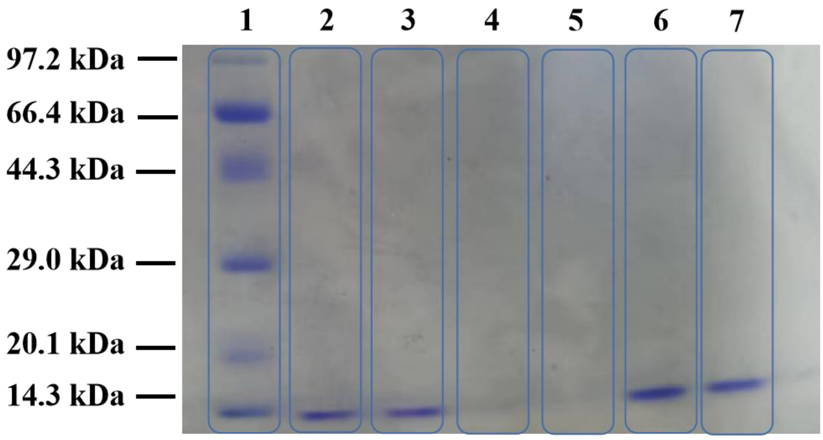

Figure S17.. Polyacrylamide gel electrophoresis (SDS-PAGE) results: lane 1: Marker, lane 2: lysozyme , lane 3: lysozyme-SH, lane 4: G-2, lane 5: h_2P(DEGMA-*co*-OEGMA)-*star*-PDSMA$_{48}$, lane 6: lysozyme + DTT, lane 7: G-2 + DTT

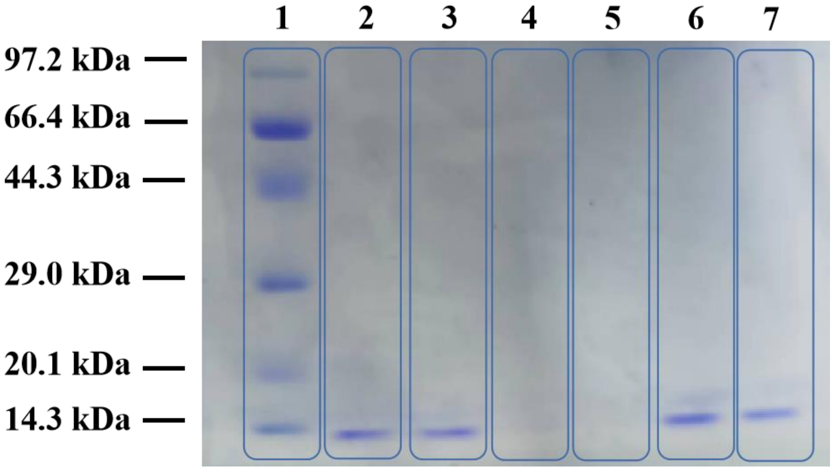

Figure S18. Polyacrylamide gel electrophoresis (SDS-PAGE) results: lane 1: Marker, lane 2: lysozyme , lane 3: lysozyme-SH, lane 4: G-3, lane 5: h_3P(DEGMA-*co*-OEGMA)-*star*-$PDSMA_{54}$, lane 6: lysozyme + DTT, lane 7: G-3 + DTT
